# The Treatment of Chronic Eczema of Children

**Published:** 1911-06

**Authors:** 

**Affiliations:** St. Joseph, Mo.


For Texas Medical Journal.

### The Treatment of Chronic Eczema of Children.

BY DR. WENZEL, ST. JOSEPH, MO.

It is often very difficult to distinguish between acute, subacute and chronic eczema, as its different stages merge into each other, yet it is safe to say that a lesion lasting for several months continuously can be considered chronic and treated accordingly.

Chronic vesicular cases of eczema with or without formation of crusts are not seen very often, yet where they exist they are of a tantalizing nature. Oftener we find seborrhoic eczemas with formation of scabs upon a reddened and weeping surface. Here it is our first task to allay the inflammation of the skin by means of a mild, bland application as, for instance, zinc ointment, Lapar's paste or a 2 per cent boric acid ointment. After reducing the inflammation an antiseborrhoic remedy should be applied. The best in this line we have is sulphur. This can be prescribed as a paste in the following way:

℞ Sulphur......................... gr. xv to xxx

Pulv. amyli......................... ʒss

Unt. zinci benz......................... ʒss

M. Sig.: Apply morning and evening.

Every other day the surface should be cleansed with benzine. Gradually, this paste can be superseded by a mild salicylic acid-sulphur ointment, as

℞ Salicylic acid......................... gr. ii to v
Sulphur prec......................... gr. x to xx
Ungt. zinci benz.
Nafthalan ......................... aa ʒss

M. Sig.: Apply twice a day.

Sometimes a delicate skin will not tolerate sulphur; then it is advisable to use the salicylic acid salve alone and add later about one-half to one per cent of ichthyol or two per cent of thigenol. After treating the eczema for a week or so, some tar should be added to accomplish a complete cure.

In the treatment of squamous cases, it is of advantage to commence first with the same ointment and simply add a little sulphur and tar. A favorite prescription of mine is the following:

℞ Sulphur prec.

Ol cade.................................aa ʒss

Pasta zinci salicylata.........................℥i

M. Sig.: Apply twice a day.

In very chronic lesions with considerable thickening of the epidermis, I commence the treatment with a 10 per cent salicylic acid salve or use the salicylated soap plaster, in order to soften the sclerotic patches, and then apply a solution of tar in alcohol and ether of about 10 to 50 per cent strength. After this application has dried, I cover the patch with Lapar's paste and keep it in place by means of a bandage. In bathing the children, it is absolutely necessary to protect the lesions by some mild paste, and we should be careful in selecting a mild soap in order not to irritate the tender skin.

Regulation of diet and proper hygienic measures are of the utmost importance.

